# Supplementary material for: Effects of Extraction Temperature of Protein from Date Palm Pollen on the Astringency Taste of Tea
Source: Foods. 2025 Feb 5;14(3):508. doi: 10.3390/foods14030508 (PMC11817395; doi:10.3390/foods14030508)
Supplement: Supplementary file 1 [file foods-14-00508-s001.zip › foods-3438494-supplementary.pdf]

Supplementary materials

Effects of Extraction Temperature of Protein from Date Palm Pollen on the Astringency Taste of Tea

Rania Mohamed [1,2,3], Xie Jizhou [1,3,4], Wei Fang [1,3,4], Luo Liyong [1,3,4], Luo Wei [1,3,4,*] and Liang Zeng [1,3,4,*]

[1] Integrative Science Center of Germplasm Creation in Western China (CHONGQING), Science City/College of Food Science, Southwest University, Chongqing 400715, China
[2] Department of Food Science and Technology, Faculty of Agriculture, University of Khartoum, Shambat 13314, Sudan
[3] Chongqing Tea Technology and Innovation Center, Chongqing, China
[4] Chongqing Key Laboratory of Speciality Food Co-Built by Sichuan and Chongqing, Chongqing 400715, China
* Correspondence: luowei1900@swu.edu.cn (L.W.); zengliangbaby@126.com (L.Z.); Tel.: +86-2368251298 (L.W.); Tel.: +86-13594172686 (L.Z.)

Table S1. The QDA Descriptive sensory evaluation of TP/DPP samples.

code	30°C Astringency	80°CAstringency	30°C Bitterness	80°C Bitterness
TP (0)	2.830 ± 0.028^{b}	3.250 ± 0.075^{a}	1.235 ± 0.023^{b}	1.155 ± 0.012^{a}
TP-DPP (0.18)	2.805 ± 0.045^{b}	2.425 ± 0.025^{a}	1.050 ± 0.028^{b}	0.995 ± 0.021^{a}
TP-DPP (0.27)	3.020 ± 0.017^{b}	2.160 ± 0.049^{a}	1.275 ± 0.052^{b}	0.990 ± 0.041^{a}
TP-DPP (0.36)	3.290 ± 0.038^{b}	2.010 ± 0.038^{a}	0.995 ± 0.078^{b}	0.805 ± 0.078^{a}
TP-DPP (0.45)	2.300 ± 0.038^{b}	1.685 ± 0.064^{a}	0.880 ± 0.075^{b}	0.735 ± 0.016^{a}

Means with the different superscript letters within the different columns are significantly different ($P < 0.05$).

Table S2. The particle size of the aggregates of EGCG/ DPP by DLS

EGCG Concentration (μM)	Particle size of sample at 30°C	Particle size of sample at 80°C
100	240.67 ± 24.85	229.00 ± 38.59
200	272.00 ± 13.08	274.33 ± 14.57
300	380.00 ± 11.36	402.00 ± 10.54
400	639.33 ± 19.09	716.00 ± 16.70

Table S3. The quenching mechanisms between EGCG and DPP by the UV-vis absorption spectroscopy in the presence and absence of EGCG.

nm	30°C-0	30°C-1	30°C-2	30°C-3	30°C-4	30°C-5	80°C-0	80°C-1	80°C-2	80°C-3	80°C-4	80°C-5
400	0.056	0.089	0.103	0.051	0.057	0.054	0.09	0.1	0.088	0.081	0.078	0.093
399	0.057	0.09	0.103	0.052	0.057	0.055	0.091	0.101	0.088	0.082	0.078	0.093
398	0.058	0.091	0.104	0.053	0.057	0.055	0.092	0.102	0.089	0.083	0.079	0.094
397	0.058	0.091	0.105	0.054	0.059	0.056	0.093	0.103	0.091	0.084	0.08	0.095
396	0.059	0.092	0.106	0.054	0.059	0.057	0.093	0.104	0.091	0.085	0.081	0.096
395	0.06	0.092	0.106	0.055	0.06	0.058	0.094	0.104	0.092	0.085	0.082	0.097

394	0.06	0.094	0.107	0.056	0.061	0.059	0.095	0.105	0.093	0.086	0.083	0.098
393	0.061	0.094	0.107	0.057	0.062	0.06	0.096	0.107	0.095	0.087	0.084	0.099
392	0.062	0.095	0.108	0.058	0.063	0.061	0.097	0.107	0.095	0.088	0.085	0.1
391	0.063	0.096	0.109	0.058	0.063	0.061	0.097	0.108	0.096	0.089	0.085	0.101
390	0.064	0.097	0.11	0.059	0.064	0.063	0.098	0.109	0.098	0.091	0.087	0.102
389	0.064	0.097	0.111	0.06	0.065	0.063	0.099	0.109	0.098	0.091	0.087	0.103
388	0.065	0.098	0.112	0.061	0.065	0.063	0.1	0.111	0.099	0.092	0.088	0.104
387	0.066	0.098	0.112	0.062	0.066	0.064	0.101	0.111	0.1	0.093	0.089	0.104
386	0.067	0.1	0.114	0.063	0.067	0.065	0.102	0.113	0.101	0.095	0.09	0.106
385	0.067	0.1	0.114	0.064	0.068	0.066	0.103	0.113	0.102	0.096	0.092	0.107
384	0.068	0.101	0.115	0.064	0.069	0.067	0.103	0.114	0.103	0.097	0.092	0.108
383	0.069	0.102	0.116	0.066	0.07	0.068	0.105	0.116	0.105	0.098	0.094	0.11
382	0.07	0.103	0.117	0.066	0.071	0.069	0.106	0.117	0.106	0.099	0.095	0.111
381	0.071	0.103	0.117	0.067	0.071	0.07	0.107	0.118	0.107	0.1	0.096	0.111
380	0.072	0.104	0.118	0.068	0.072	0.071	0.107	0.119	0.108	0.101	0.097	0.112
379	0.073	0.106	0.12	0.069	0.074	0.072	0.109	0.12	0.11	0.103	0.099	0.114
378	0.074	0.107	0.121	0.07	0.074	0.073	0.11	0.121	0.111	0.104	0.1	0.115
377	0.074	0.107	0.121	0.071	0.075	0.074	0.111	0.122	0.112	0.105	0.1	0.116
376	0.076	0.109	0.122	0.072	0.076	0.075	0.112	0.123	0.113	0.106	0.102	0.118
375	0.077	0.109	0.123	0.073	0.078	0.076	0.113	0.124	0.115	0.108	0.103	0.12
374	0.077	0.11	0.124	0.074	0.078	0.076	0.114	0.125	0.116	0.108	0.104	0.12
373	0.078	0.111	0.125	0.075	0.079	0.078	0.115	0.127	0.118	0.11	0.105	0.121
372	0.079	0.111	0.126	0.076	0.081	0.078	0.116	0.128	0.118	0.111	0.107	0.123
371	0.081	0.113	0.127	0.077	0.082	0.08	0.118	0.13	0.12	0.113	0.109	0.124
370	0.082	0.114	0.128	0.078	0.082	0.081	0.119	0.131	0.122	0.114	0.11	0.126
369	0.083	0.115	0.13	0.079	0.083	0.082	0.12	0.132	0.123	0.115	0.111	0.127
368	0.083	0.116	0.13	0.081	0.084	0.083	0.121	0.133	0.124	0.117	0.112	0.128
367	0.085	0.116	0.131	0.082	0.086	0.084	0.122	0.134	0.126	0.118	0.114	0.13
366	0.086	0.119	0.133	0.083	0.087	0.086	0.124	0.136	0.128	0.12	0.116	0.132
365	0.087	0.12	0.134	0.084	0.088	0.087	0.126	0.138	0.129	0.121	0.117	0.134
364	0.088	0.12	0.135	0.086	0.089	0.088	0.126	0.139	0.131	0.123	0.118	0.135
363	0.089	0.122	0.136	0.087	0.09	0.09	0.127	0.14	0.132	0.124	0.12	0.136
362	0.091	0.123	0.138	0.088	0.092	0.09	0.129	0.142	0.134	0.126	0.121	0.138
361	0.091	0.124	0.138	0.089	0.093	0.091	0.13	0.143	0.135	0.127	0.123	0.139
360	0.093	0.125	0.14	0.09	0.094	0.093	0.131	0.145	0.137	0.129	0.125	0.141
359	0.094	0.126	0.141	0.091	0.095	0.094	0.133	0.146	0.139	0.131	0.126	0.143
358	0.095	0.127	0.142	0.093	0.097	0.096	0.135	0.148	0.141	0.133	0.128	0.146
357	0.097	0.13	0.144	0.095	0.098	0.098	0.136	0.15	0.143	0.135	0.131	0.148
356	0.098	0.131	0.146	0.097	0.1	0.099	0.138	0.152	0.145	0.137	0.132	0.15
355	0.099	0.132	0.147	0.098	0.101	0.1	0.139	0.153	0.146	0.139	0.134	0.152
354	0.101	0.133	0.148	0.099	0.103	0.102	0.141	0.155	0.148	0.141	0.136	0.154
353	0.102	0.135	0.15	0.101	0.104	0.104	0.143	0.157	0.151	0.143	0.138	0.156
352	0.104	0.136	0.152	0.103	0.106	0.105	0.144	0.159	0.153	0.145	0.14	0.159
351	0.105	0.137	0.153	0.104	0.107	0.107	0.145	0.161	0.155	0.147	0.142	0.161
350	0.106	0.139	0.154	0.106	0.109	0.109	0.147	0.163	0.158	0.149	0.144	0.163
349	0.108	0.141	0.156	0.108	0.111	0.111	0.15	0.165	0.16	0.152	0.147	0.166

348	0.109	0.142	0.158	0.109	0.112	0.112	0.15	0.167	0.162	0.154	0.149	0.169
347	0.111	0.144	0.159	0.111	0.114	0.114	0.153	0.17	0.165	0.157	0.152	0.171
346	0.113	0.146	0.162	0.113	0.116	0.117	0.155	0.172	0.168	0.16	0.155	0.174
345	0.114	0.147	0.163	0.115	0.118	0.118	0.156	0.174	0.17	0.162	0.157	0.177
344	0.116	0.149	0.165	0.117	0.119	0.12	0.158	0.176	0.172	0.165	0.159	0.18
343	0.118	0.151	0.167	0.119	0.121	0.122	0.16	0.178	0.175	0.167	0.162	0.183
342	0.119	0.152	0.169	0.12	0.123	0.125	0.162	0.181	0.178	0.17	0.165	0.186
341	0.121	0.154	0.17	0.122	0.125	0.126	0.164	0.183	0.18	0.173	0.167	0.189
340	0.122	0.156	0.172	0.124	0.127	0.128	0.165	0.185	0.183	0.176	0.17	0.192
339	0.124	0.157	0.174	0.126	0.128	0.13	0.167	0.188	0.186	0.178	0.173	0.195
338	0.126	0.159	0.176	0.128	0.131	0.133	0.169	0.191	0.189	0.181	0.176	0.198
337	0.127	0.162	0.177	0.13	0.133	0.134	0.172	0.193	0.191	0.184	0.179	0.201
336	0.129	0.163	0.18	0.133	0.135	0.137	0.173	0.196	0.194	0.187	0.182	0.205
335	0.131	0.165	0.182	0.135	0.137	0.139	0.175	0.198	0.197	0.189	0.184	0.208
334	0.132	0.167	0.183	0.137	0.139	0.142	0.177	0.201	0.2	0.192	0.187	0.212
333	0.134	0.169	0.186	0.139	0.141	0.145	0.18	0.203	0.203	0.195	0.191	0.215
332	0.136	0.171	0.188	0.141	0.144	0.148	0.182	0.206	0.206	0.198	0.193	0.219
331	0.137	0.173	0.19	0.143	0.146	0.15	0.183	0.208	0.209	0.201	0.196	0.222
330	0.139	0.175	0.193	0.146	0.149	0.153	0.186	0.211	0.212	0.204	0.199	0.226
329	0.141	0.177	0.195	0.148	0.151	0.156	0.187	0.214	0.216	0.208	0.203	0.23
328	0.143	0.18	0.198	0.151	0.154	0.16	0.19	0.217	0.219	0.211	0.207	0.234
327	0.145	0.182	0.201	0.154	0.157	0.164	0.192	0.22	0.223	0.215	0.21	0.239
326	0.146	0.184	0.203	0.157	0.16	0.167	0.194	0.223	0.226	0.218	0.214	0.244
325	0.148	0.187	0.206	0.161	0.164	0.172	0.196	0.226	0.23	0.223	0.218	0.249
324	0.149	0.19	0.209	0.164	0.167	0.177	0.198	0.23	0.234	0.226	0.222	0.255
323	0.151	0.193	0.213	0.168	0.171	0.183	0.2	0.233	0.238	0.231	0.227	0.261
322	0.153	0.196	0.217	0.172	0.175	0.189	0.202	0.237	0.243	0.236	0.231	0.268
321	0.155	0.2	0.221	0.176	0.18	0.196	0.205	0.241	0.248	0.241	0.237	0.275
320	0.157	0.203	0.226	0.181	0.186	0.203	0.207	0.246	0.253	0.246	0.243	0.284
319	0.158	0.208	0.231	0.186	0.191	0.212	0.209	0.25	0.259	0.252	0.249	0.293
318	0.16	0.213	0.237	0.193	0.198	0.222	0.212	0.255	0.265	0.259	0.256	0.303
317	0.162	0.217	0.243	0.2	0.205	0.232	0.213	0.261	0.272	0.266	0.263	0.314
316	0.164	0.223	0.25	0.206	0.213	0.245	0.216	0.267	0.28	0.274	0.272	0.327
315	0.165	0.229	0.257	0.214	0.221	0.257	0.218	0.273	0.288	0.282	0.28	0.34
314	0.168	0.235	0.266	0.223	0.231	0.272	0.221	0.28	0.297	0.292	0.291	0.354
313	0.17	0.241	0.273	0.232	0.24	0.287	0.223	0.287	0.306	0.301	0.3	0.369
312	0.172	0.248	0.283	0.241	0.251	0.303	0.226	0.294	0.315	0.312	0.311	0.387
311	0.173	0.255	0.292	0.251	0.261	0.32	0.228	0.302	0.325	0.322	0.322	0.403
310	0.175	0.263	0.302	0.261	0.272	0.338	0.23	0.309	0.335	0.332	0.334	0.422
309	0.177	0.271	0.313	0.272	0.285	0.357	0.233	0.318	0.347	0.344	0.346	0.441
308	0.179	0.28	0.324	0.283	0.298	0.377	0.235	0.327	0.358	0.356	0.359	0.462
307	0.181	0.288	0.335	0.295	0.311	0.398	0.239	0.336	0.37	0.369	0.373	0.482
306	0.183	0.297	0.346	0.307	0.324	0.419	0.241	0.345	0.382	0.381	0.386	0.503
305	0.185	0.306	0.358	0.319	0.338	0.44	0.244	0.354	0.394	0.394	0.4	0.525
304	0.187	0.315	0.37	0.332	0.351	0.462	0.246	0.364	0.407	0.407	0.414	0.547
303	0.19	0.325	0.382	0.345	0.365	0.484	0.249	0.373	0.419	0.421	0.428	0.568

302	0.192	0.333	0.393	0.357	0.379	0.505	0.252	0.382	0.431	0.433	0.442	0.59
301	0.194	0.343	0.405	0.369	0.393	0.527	0.255	0.392	0.444	0.447	0.456	0.612
300	0.196	0.352	0.417	0.382	0.407	0.548	0.257	0.401	0.456	0.459	0.469	0.634
299	0.199	0.361	0.429	0.393	0.42	0.569	0.26	0.41	0.468	0.472	0.483	0.655
298	0.201	0.37	0.441	0.406	0.434	0.591	0.263	0.42	0.48	0.485	0.497	0.677
297	0.204	0.379	0.453	0.419	0.448	0.613	0.266	0.43	0.493	0.499	0.511	0.699
296	0.206	0.388	0.465	0.431	0.462	0.634	0.269	0.439	0.506	0.512	0.525	0.72
295	0.209	0.398	0.477	0.444	0.476	0.656	0.273	0.449	0.518	0.525	0.54	0.742
294	0.212	0.408	0.489	0.457	0.49	0.677	0.276	0.458	0.531	0.539	0.554	0.763
293	0.215	0.417	0.501	0.469	0.503	0.698	0.28	0.469	0.544	0.552	0.568	0.785
292	0.218	0.426	0.513	0.481	0.516	0.719	0.283	0.478	0.556	0.565	0.581	0.806
291	0.221	0.436	0.525	0.494	0.531	0.74	0.287	0.488	0.569	0.579	0.596	0.828
290	0.224	0.445	0.537	0.506	0.544	0.761	0.291	0.498	0.582	0.592	0.61	0.849
289	0.227	0.455	0.549	0.519	0.558	0.782	0.295	0.509	0.595	0.605	0.625	0.871
288	0.23	0.465	0.562	0.533	0.573	0.805	0.299	0.519	0.608	0.62	0.64	0.894
287	0.234	0.475	0.575	0.546	0.588	0.827	0.303	0.53	0.622	0.634	0.655	0.917
286	0.237	0.485	0.588	0.56	0.603	0.851	0.307	0.541	0.637	0.649	0.67	0.941
285	0.24	0.495	0.601	0.574	0.619	0.875	0.31	0.552	0.65	0.664	0.687	0.965
284	0.243	0.506	0.615	0.589	0.634	0.9	0.314	0.563	0.666	0.68	0.703	0.991
283	0.245	0.517	0.629	0.603	0.651	0.926	0.317	0.575	0.68	0.695	0.72	1.018
282	0.247	0.528	0.644	0.618	0.667	0.952	0.32	0.586	0.696	0.711	0.737	1.044
281	0.249	0.538	0.657	0.633	0.683	0.978	0.323	0.597	0.711	0.727	0.755	1.071
280	0.251	0.548	0.671	0.646	0.699	1.003	0.325	0.608	0.724	0.742	0.77	1.097
279	0.252	0.557	0.682	0.658	0.712	1.024	0.326	0.616	0.737	0.755	0.784	1.119
278	0.254	0.564	0.691	0.669	0.724	1.043	0.329	0.625	0.747	0.766	0.796	1.137
277	0.255	0.569	0.698	0.676	0.732	1.055	0.331	0.63	0.755	0.774	0.805	1.151
276	0.256	0.573	0.704	0.682	0.739	1.066	0.333	0.635	0.761	0.78	0.812	1.161
275	0.257	0.577	0.708	0.686	0.744	1.074	0.334	0.639	0.766	0.785	0.817	1.17
274	0.259	0.58	0.711	0.69	0.748	1.08	0.336	0.642	0.77	0.79	0.821	1.176
273	0.259	0.58	0.713	0.691	0.75	1.082	0.336	0.643	0.772	0.792	0.824	1.179
272	0.26	0.581	0.713	0.692	0.749	1.083	0.337	0.644	0.773	0.793	0.823	1.18
271	0.26	0.579	0.71	0.69	0.746	1.078	0.337	0.642	0.771	0.791	0.822	1.176
270	0.261	0.577	0.707	0.686	0.742	1.071	0.338	0.641	0.768	0.787	0.818	1.169
269	0.261	0.572	0.701	0.68	0.736	1.059	0.338	0.637	0.762	0.781	0.811	1.158
268	0.261	0.567	0.693	0.672	0.727	1.044	0.338	0.631	0.754	0.773	0.802	1.142
267	0.262	0.56	0.684	0.663	0.715	1.025	0.338	0.625	0.746	0.763	0.791	1.123
266	0.262	0.552	0.673	0.651	0.702	1.003	0.338	0.618	0.735	0.752	0.779	1.102
265	0.262	0.544	0.661	0.639	0.688	0.98	0.338	0.609	0.724	0.74	0.765	1.078
264	0.263	0.535	0.648	0.626	0.674	0.955	0.338	0.6	0.712	0.726	0.751	1.054
263	0.263	0.525	0.635	0.612	0.658	0.928	0.338	0.591	0.699	0.712	0.735	1.027
262	0.263	0.516	0.621	0.599	0.641	0.902	0.338	0.581	0.685	0.698	0.72	1.001
261	0.264	0.506	0.607	0.584	0.624	0.874	0.338	0.571	0.672	0.683	0.703	0.974
260	0.265	0.496	0.594	0.57	0.608	0.846	0.338	0.562	0.659	0.669	0.687	0.947
259	0.265	0.486	0.58	0.556	0.592	0.819	0.338	0.552	0.646	0.654	0.671	0.919
258	0.266	0.477	0.567	0.543	0.576	0.791	0.339	0.543	0.633	0.64	0.655	0.892
257	0.267	0.468	0.554	0.529	0.56	0.764	0.339	0.534	0.62	0.627	0.64	0.866

256	0.268	0.459	0.541	0.516	0.546	0.739	0.34	0.525	0.609	0.614	0.626	0.841
255	0.27	0.451	0.53	0.505	0.532	0.716	0.341	0.518	0.598	0.602	0.613	0.818
254	0.271	0.444	0.519	0.494	0.519	0.694	0.342	0.512	0.588	0.591	0.601	0.796
253	0.273	0.438	0.51	0.485	0.508	0.674	0.343	0.506	0.58	0.582	0.591	0.777
252	0.276	0.433	0.503	0.478	0.499	0.658	0.345	0.502	0.574	0.575	0.583	0.762
251	0.278	0.43	0.498	0.472	0.492	0.645	0.347	0.5	0.569	0.57	0.577	0.75
250	0.281	0.429	0.494	0.469	0.487	0.636	0.35	0.498	0.567	0.568	0.573	0.742
249	0.284	0.428	0.493	0.468	0.486	0.631	0.353	0.499	0.568	0.568	0.573	0.739
248	0.287	0.431	0.495	0.47	0.487	0.631	0.356	0.503	0.571	0.571	0.576	0.74
247	0.292	0.435	0.501	0.476	0.493	0.638	0.361	0.509	0.578	0.578	0.583	0.749
246	0.296	0.444	0.51	0.485	0.503	0.651	0.366	0.518	0.589	0.589	0.595	0.764
245	0.301	0.454	0.522	0.499	0.517	0.671	0.371	0.53	0.603	0.604	0.611	0.787
244	0.307	0.468	0.54	0.516	0.536	0.7	0.378	0.546	0.623	0.625	0.632	0.818
243	0.313	0.486	0.562	0.54	0.561	0.739	0.384	0.566	0.648	0.652	0.66	0.859
242	0.32	0.507	0.589	0.568	0.592	0.785	0.392	0.588	0.677	0.682	0.692	0.907
241	0.327	0.532	0.621	0.601	0.628	0.841	0.401	0.615	0.71	0.718	0.73	0.966
240	0.335	0.561	0.658	0.64	0.671	0.906	0.41	0.646	0.75	0.76	0.776	1.035
239	0.344	0.594	0.701	0.685	0.721	0.982	0.42	0.681	0.796	0.809	0.827	1.114
238	0.354	0.633	0.75	0.736	0.776	1.068	0.433	0.721	0.847	0.863	0.884	1.201
237	0.366	0.675	0.804	0.792	0.838	1.164	0.446	0.765	0.903	0.922	0.948	1.3
236	0.379	0.721	0.863	0.853	0.906	1.269	0.46	0.812	0.965	0.988	1.017	1.405
235	0.392	0.77	0.927	0.92	0.979	1.38	0.476	0.864	1.031	1.057	1.091	1.519
234	0.407	0.824	0.995	0.991	1.057	1.501	0.493	0.919	1.102	1.131	1.171	1.641
233	0.423	0.881	1.067	1.065	1.139	1.624	0.511	0.977	1.175	1.21	1.255	1.77
232	0.44	0.94	1.142	1.143	1.225	1.756	0.529	1.037	1.254	1.292	1.343	1.902
231	0.457	1.002	1.222	1.227	1.317	1.893	0.55	1.101	1.336	1.379	1.435	2.04
230	0.473	1.067	1.306	1.314	1.413	2.037	0.57	1.168	1.422	1.473	1.532	2.186
229	0.492	1.136	1.395	1.406	1.514	2.188	0.592	1.24	1.514	1.566	1.636	2.33
228	0.51	1.21	1.489	1.504	1.62	2.341	0.614	1.314	1.61	1.668	1.745	2.486
227	0.528	1.285	1.586	1.604	1.731	2.5	0.637	1.393	1.71	1.776	1.856	2.627
226	0.547	1.361	1.684	1.706	1.845	2.646	0.659	1.474	1.813	1.884	1.972	2.882
225	0.566	1.44	1.784	1.811	1.956	2.837	0.682	1.555	1.916	1.99	2.083	3.13
224	0.585	1.518	1.883	1.911	2.067	3.067	0.705	1.635	2.016	2.095	2.193	3.287
223	0.604	1.596	1.98	2.009	2.17	3.26	0.728	1.713	2.114	2.192	2.307	3.532
222	0.623	1.668	2.066	2.1	2.272	3.334	0.751	1.787	2.207	2.292	2.398	3.586
221	0.643	1.742	2.157	2.191	2.364	3.429	0.775	1.865	2.298	2.379	2.486	3.589
220	0.665	1.819	2.242	2.283	2.458	3.484	0.802	1.941	2.382	2.47	2.565	3.655
219	0.687	1.895	2.34	2.373	2.537	3.589	0.827	2.024	2.471	2.56	2.66	3.715
218	0.711	1.978	2.426	2.46	2.624	3.594	0.855	2.105	2.56	2.651	2.787	3.719
217	0.735	2.061	2.511	2.546	2.702	3.54	0.883	2.19	2.643	2.723	2.911	3.656
216	0.761	2.155	2.601	2.624	2.832	3.478	0.914	2.279	2.742	2.882	3.018	3.645
215	0.789	2.25	2.671	2.702	2.937	3.432	0.946	2.371	2.86	2.992	3.149	3.533
214	0.819	2.345	2.728	2.77	2.962	3.337	0.98	2.458	2.962	3.019	3.147	3.428
213	0.85	2.442	2.891	2.891	3.028	3.307	1.015	2.543	3	3.09	3.157	3.393
212	0.885	2.517	2.889	2.888	2.996	3.262	1.053	2.612	3.026	3.086	3.119	3.387
211	0.919	2.572	2.909	2.883	2.935	3.118	1.09	2.662	2.99	3.019	3.049	3.254

210	0.957	2.602	2.859	2.812	2.885	3.049	1.132	2.68	2.958	2.937	3.015	3.146
209	0.997	2.628	2.814	2.77	2.835	3.018	1.176	2.683	2.913	2.914	2.94	3.147
208	1.041	2.6	2.722	2.679	2.722	2.9	1.221	2.669	2.827	2.828	2.833	2.986
207	1.089	2.575	2.677	2.649	2.676	2.8	1.267	2.631	2.714	2.735	2.714	2.922
206	1.14	2.567	2.632	2.607	2.631	2.717	1.319	2.606	2.668	2.685	2.688	2.826
205	1.193	2.509	2.587	2.55	2.57	2.663	1.369	2.563	2.625	2.609	2.618	2.725
204	1.248	2.474	2.539	2.504	2.531	2.606	1.427	2.51	2.56	2.574	2.574	2.659
203	1.309	2.434	2.5	2.474	2.487	2.575	1.481	2.48	2.532	2.539	2.546	2.601
202	1.366	2.377	2.439	2.412	2.439	2.499	1.534	2.432	2.473	2.467	2.478	2.554
201	1.424	2.333	2.389	2.369	2.384	2.448	1.581	2.371	2.419	2.431	2.431	2.511
200	1.48	2.288	2.352	2.321	2.33	2.407	1.629	2.322	2.371	2.376	2.387	2.475
199	1.528	2.235	2.288	2.268	2.28	2.342	1.675	2.275	2.321	2.339	2.326	2.409
198	1.57	2.18	2.23	2.199	2.231	2.304	1.706	2.216	2.266	2.263	2.264	2.345
197	1.6	2.116	2.167	2.138	2.16	2.22	1.725	2.154	2.204	2.208	2.206	2.296
196	1.608	2.058	2.107	2.073	2.089	2.163	1.726	2.08	2.121	2.135	2.124	2.231
195	1.591	1.96	2.014	1.998	2.008	2.067	1.7	2.005	2.047	2.049	2.049	2.131
194	1.564	1.874	1.94	1.894	1.913	1.982	1.658	1.902	1.946	1.972	1.967	2.031
193	1.499	1.75	1.808	1.779	1.792	1.844	1.566	1.789	1.833	1.815	1.844	1.917
192	1.393	1.614	1.656	1.639	1.655	1.718	1.457	1.655	1.704	1.685	1.707	1.778
191	1.257	1.462	1.514	1.484	1.496	1.562	1.334	1.49	1.536	1.533	1.548	1.629
190	1.093	1.265	1.331	1.289	1.303	1.376	1.154	1.307	1.346	1.358	1.353	1.439

30°C and 80°C means DPP extraction temperatures

0,1,2,3,4,5 means different concentrations of EGCG
